# Wound healing: time to look for intelligent, ‘natural’ immunological approaches?

**DOI:** 10.1186/s12865-017-0207-y

**Published:** 2017-06-21

**Authors:** Olivier Garraud, Wael N. Hozzein, Gamal Badr

**Affiliations:** 1 GIMAP-EA3064, Faculty of medicine of Saint-Etienne, University of Lyon, 42023 Saint-Etienne, France; 2 National Institute for Blood Transfusion, 75015 Paris, France; 3 Bioproducts Research Chair, Department of Zoology, College of Science, King Saud University, Riyadh, Saudi Arabia; 4 Botany Department, Faculty of Science, Beni-Suef University, Beni-Suef, Egypt; 5 Laboratory of Immunology and Molecular Physiology, Zoology Department, Faculty of Science, Assiut University, 71516 Assiut, Egypt

**Keywords:** Wound, Diabetes, Healing factors, Platelets, Whey proteins, Propolis, Bee venom

## Abstract

There is now good evidence that cytokines and growth factors are key factors in tissue repair and often exert anti-infective activities. However, engineering such factors for global use, even in the most remote places, is not realistic. Instead, we propose to examine how such factors work and to evaluate the reparative tools generously provided by ‘nature.’ We used two approaches to address these objectives. The first approach was to reappraise the internal capacity of the factors contributing the most to healing in the body, i.e., blood platelets. The second was to revisit natural agents such as whey proteins, (honey) bee venom and propolis. The platelet approach elucidates the inflammation spectrum from physiology to pathology, whereas milk and honey derivatives accelerate diabetic wound healing. Thus, this review aims at offering a fresh view of how wound healing can be addressed by natural means.

## Background

Wounds occur on multiple occasions during one’s lifetime. Although they are less visible than external wounds, internal wounds frequently occur at the vascular surface. Such vascular attrition must be repaired to avoid bleeding and fluid loss from the vessels to tissues. The mucosae are also exposed to wounds that occasionally result in bleeding and/or infection. Additionally, organs can suffer wounds caused by external insults (weapons, surgery) or internal disorders (disease). Overall, wounds may be clear (surgical) or complex, clean or soiled, and aseptic or septic. Wounds may occur occasionally or frequently (recurrent) and may be acute or chronic. Chronic wounds are often associated with a patient’s inability to sufficiently (correctly) heal and clear the source or cause of the attrition, such as in chronic infections or metabolic disorders. Metabolic disorders such as diabetes, which can result in infections and chronic wounds, are multifactorial. It has been estimated that nearly 9% of the global population is affected by diabetes, and diabetes is a leading cause of a chronic type of wound with vascular participation that is often infected and exhibits poor healing [1, 2]. Thus, with an estimated worldwide population of 7.3 billion, nearly one billion people are likely to suffer acute and/or chronic wounds (in the USA, 6.5 million people suffer from chronic wounds) [3]. Therefore, for multiple reasons, hundreds of individuals present with skin/tissue attrition and hundreds more have organ lesions. Poverty, poor sanitation, malnutrition, insect bites, and envenomation, may either cause wounds or aggravate their consequences [4].

**Fig. 1:**
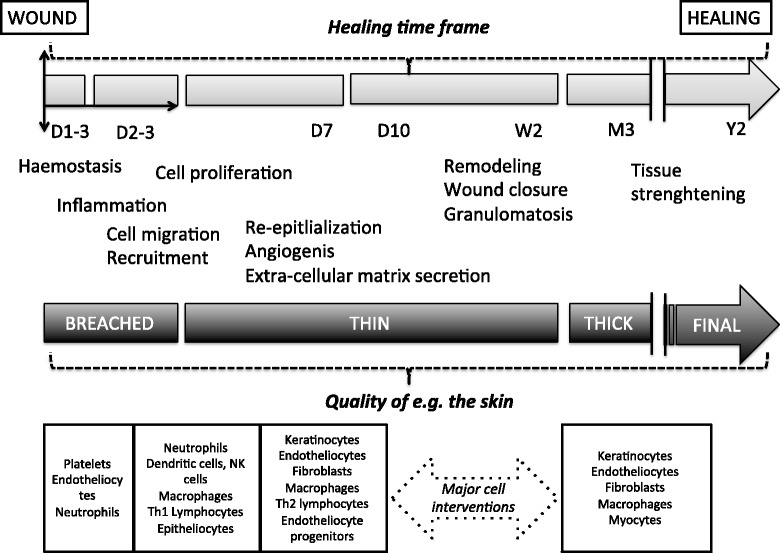
Cartoons—from top to bottom—: i) The healing time frame expressed in days (D), weeks (W) and years (Y); ii) The main cell ant tissue events; iii) the quality of the skin; iv) the major cell and cell subsets involved (partly inspired by Ref [84])

Healing is a complex process that evolves in phases and involves numerous key cellular and molecular players and frequently, because many wounds create breaches of the microvasculature, coagulation effectors [5]. Inflammation is now regarded as an initiator of healing, as long as the inflammation is contained and does not escape regulatory mechanisms and become pathologic. Wound repair is classically viewed as a four-step process: i) hemostasis; ii) inflammation; iii) cell proliferation/granulation; and iv) remodeling/maturation. The time frames for each step in the process are 1–3 days, 3–20 days, 7–40 days and 40 days to 2 years, respectively, with overlaps in sub-processes. If a wound is cutaneous, after the bleeding has stopped, the skin is temporarily thin, then consists of stronger, tightening skin tissue; in the final phase, the skin tissue is restored (a schematic of the process is presented in Fig. 1). Table 1 further presents the major cellular and molecular actors in the healing stages.

**Table 1:** Molecular factor essential to healing [84–87]

CYTOKINES, CHEMOKINES, GROWTH FACTORS - IL-8 - SDF-1 - VEGF-α - PDGF - MCP-1 - IGF-1 - EGF-1 - Keratinocyte growth factor - Angiopoietin-1 - MIP-1 - α-, β-erythropoietin - GM-CSF - FGF-2	MATRIX GLYCOPROTEINS - Collagen - Fibrin - Fibronectin - Vitronectin -Tenascin
	ENZYMES and INHIBITORS - α2-macroglobulin - MMP-1,2,3,9 - TIMPs - ACE - Serine Proteases : urokine-type plasminogen activator - Cathepsin G, - Neutrophil elastase - NO antagonists
OTHERS - Complement factors	

Importantly, because conventional health care is not accessible everywhere or, when it is, is not made available to everyone because of the cost, the majority of the world’s population commonly resorts to traditional medicines with various outcomes and often at the expense of imposing a superinfection. There is now good evidence that cytokines and growth factors are key actors in tissue repair and often exert anti-infective activities. However, engineering such factors for global use, particularly in the most remote places, is not realistic. We propose instead to examine how such factors work and to evaluate the reparative tools generously provided by ‘nature.’ We used two approaches to address these objectives. The first approach was to assess the internal capacity of the factors contributing the most to healing in the body, i.e., blood platelets. The second approach was to evaluate natural agents such as whey proteins from milk (and particularly from camels), (honey) bee venom and propolis.

### Blood platelets as healing factors

Blood platelets are small elements derived from the fragmentation of megakaryocytes. Despite being non-nucleated, platelets can exert a number of essential functions [6, 7]. Platelets are primarily acknowledged as being essential to primary hemostasis and coagulation. Physiologically, platelets patrol along the vascular arborescence and repair injured vascular endothelium, which is considered a healing function [8]. The hemostatic functions of platelets (adhesion, activation, aggregation, and the recruitment of other platelets and leukocytes) are mediated by a number of specific receptors that bind to ligands on the sub-endothelial structure after vessel attrition (such as fibrinogen, collagen, vWF, etc.), on other platelets, and on leukocytes (integrins). However, the majority of such structures can also serve as ligands for numerous infectious pathogens (viruses, bacteria, parasites and fungi). Thus, platelets play a central role during tissue attrition (wounding) because they assist in hemostasis, innate immunity, anti-infection activity, inflammation and the secretion of a myriad of factors (including growth factors for stimulating the production of blood cells in bone marrow, chemokines to attract innate immune cells, reparative cytokines, and other biological response modifiers (BRMs) with a variety of functions) [9–11].

It is now understood that the essential function of platelets is to detect danger signals that are delivered by two principal types of warning systems: 1) External lesions. The archetype of this system is a vascular wound exposed to the surface (skin, muscle, and vessel), but this type also comprises sensing infectious agents with differential responses based on whether an agent is highly pathogenic, less pathogenic or not pathogenic [12, 13]; 2) Internal danger signals that activate platelets. The archetype of this system is the sensing of vascular erosion/attrition due to shear stresses inflicted by globules flowing into microscopic vessels that are half the size of such globules [14, 15]. This type of warning can also occur in several other types of tissue and organ attrition caused by a chronic infection, so-called auto-inflammatory processes, etc. [16]. When patrolling along the vasculature (platelets are essentially circulating cell elements), platelets sense danger. The first danger situation that is sensed is vascular endothelial attrition. Platelets adhere to the injured vascular endothelium, undergo activation and secrete factors that are stored in abundance within granules (these factors are derived from megakaryocytes or absorbed from the plasma and eventually are secreted by the platelets). Platelets are loaded with healing and repairing factors, such as PDGF, EGF, VEGF, TGF-ß, TF, etc., and with growth factors as well as cytokines and chemokines that mobilize bone marrow progenitor cells, which are attracted to the lesion sites and also secrete repairing factors [17–19]. Platelets have two main cell partners: endotheliocytes and leukocytes (platelets engage in very complex relationships with leukocyte subsets both physiologically and pathophysiologically; for reviews, see [9, 18, 19]). Approximately 10% of the platelet count is used daily to perform repairs and fix the pathophysiological alterations of the vascular endothelium due to shear stress inflicted by circulating cells and blood pressure.

Platelets are currently being evaluated as a therapeutic approach beyond their seminal role in assisting in hemostasis (platelet component transfusion) [20] and can be used in several forms. Autologous platelet-rich plasma is commonly injected in altered or injured tissues; regular protocols have been established to repair tendons, cartilage, bone, joints, muscle and soft tissues (including cardiac tissue), and the retina [21–25]. Platelet gels or glues come from two major sources: autologous and allogeneic [26, 27]. Platelet gels/glues are also used in sports medicine and traumatology, dental and ophthalmic surgery. Platelet preparations are also used in facial surgery, including aesthetic surgery [28]. Side effects, such as blood-borne infections, are still possible but the risk of side effects is mitigated when the platelets are autologous. When platelets are derived from blood donors, the preparations can benefit from pathogen reduction technologies (complementing labwork testing that assesses negativity for principal infectious markers in the product) [29]. The therapeutic factors in platelet preparations are the same as those in fresh platelets and are dominated by VEGF and TGF-ß. Platelet repairing factors have been proven to have healing activity along with anti-inflammatory and anti-infectious activities [30]. Experimental findings have suggested a potential use of platelets or platelet preparations for ulcerative wounds with vascular participation and infections, such as gastric ulcers [31] and diabetes-related wounds [32]. Although interesting data have been reported, and platelet therapy in treating diabetic foot ulcers is promising, more clinical trials are required.

Additionally, because platelets have hemostatic, pro-inflammatory (to initiate the late-phase angiogenesis and remodeling phases), anti-inflammatory and anti-infectious effects, these blood components or derivatives. Because of the medical and economic effects of diabetes worldwide and the easy access of platelets, especially autologous platelets, platelets may be a promising avenue for the treatment of diabetes-associated wounds. However, platelet therapy mandates that safety and quality processes be strictly followed; such adherence may not be easy to fulfill in areas where diabetes occurs very frequently but transfusions are not yet optimized, as is the case in almost every developing country. Therefore, other natural alternatives must be considered.

### Healing properties of un-denatured whey protein in diabetic models

The ability of animals to repair wounds after an injury is critical for survival [33]. A multitude of cellular events, such as cell proliferation, cell migration, contraction, extracellular matrix degradation and synthesis, must occur to achieve wound closure and the regeneration of the injured dermis [34]. These events rely on the temporal expression and activation of a variety of proteins such as growth factors, cytokines and matrix metalloproteinases [35]. The identification of dietary proteins that enhance skin repair contributes to the understanding of the wound-healing process and to therapeutic design. Whey protein is thought to be the highest quality protein available, as compared to other proteins, such as ovalbumin, casein, beef meat, or soy. Whey protein contains all the essential and non-essential amino acids and is an excellent source of glutamine and branched-chain amino acids, which are necessary for cell growth [36]. Thus, whey contains high levels of amino acids that are important for wound healing. These amino acids include arginine, glycine, and, in particular, the branch-chained amino acids leucine, isoleucine and valine, which are essential to promoting the healing of bones, skin, and muscle tissues. Another amino acid, proline, aids in the production of collagen, heals cartilage and strengthens joints, tendons and cardiac muscle.

Various spatial arrangements of amino acids in whey protein influence immune responses in different ways [36]. Therefore, it has been hypothesized that unique amino acid groups or peptides derived from whey proteins after ingestion can have significant immunomodulatory activities. Bioactive components include lactoferrin, lysozyme, lactoperoxidase, glycomacropeptide, alpha-lactalbumin, bovine serum albumin, various growth factors and immunoglobulins (Igs). Many of these components possess immunobiological properties. Lactoferrin exhibits anticancer, antiviral, antibacterial, and antifungal activity. Lactoferrin plays active roles in iron transport and in the cytotoxic defenses of neutrophils and scavenges free iron and associated oxygen radicals [37]. Un-denatured whey protein has been shown to be a potent inducer of glutathione, thereby reducing cellular damage and improving intracellular function [38]. Glutathione is a potent intracellular tripeptide that vigorously binds damaging free radical molecules that would otherwise harm the cell. During the inflammatory phase, the release of oxygen radicals by leukocytes, the subsequent lipid peroxidation of cellular and organelle membranes, the disruption of the intracellular matrix, and the alteration of important protein enzymatic processes cause tissue damage [37]. The generation of oxygen radicals is normally mitigated by the presence of adequate endogenous antioxidant defenses [37]. In diabetes, oxidative stress is increased by a system in which the rate of free radical production is enhanced and/or endogenous antioxidant mechanisms are impaired. Oxidative-stress-induced free radicals have been found to be key players in the pathophysiology of diabetes mellitus and neurodegenerative diseases [39]. Many studies have confirmed that glutathione, which is increased by dietary whey protein, is a powerful antioxidant? The efficiency of cysteine in increasing glutathione levels is greater when this amino acid is delivered as whey protein rather than as free cysteine [40–42].

Animals fed un-denatured whey protein display accelerated wound healing. Whey protein has been reported to act at the intersection of inflammation and proliferation (Fig. 1) because some components in whey protein exert mitogenic activity on lymphocytes [42]. Being rich in cysteine, whey protein seems to control lymphocyte functions; both the conventional T (Th1)-lymphocytes, which are prone to stimulate fibroblasts, and B-lymphocytes appear to be targeted by this amino acid. Regarding B-lymphocytes, at least in experimental models, whey protein influences immunoglobulin (Ig) production in the spleen [42]. B-lymphocytes are impaired in animals with experimentally induced diabetes, and experimentally restoring normal B-cell physiology has been shown to be beneficial [42, 43]. Whey protein is also a source of abundant levels of arginine, an important factor in sustaining the activity of natural killer (NK) cells, which secrete appreciable amounts of cytokines that are active in cell proliferation, such as interferon-γ.

Whey protein is able to decrease the effects of oxygen radicals and lipid peroxidation by increasing the antioxidant glutathione and, thereby, stimulating epithelialization and the proliferation of fibroblasts as well as increasing the secretion of both pre-inflammatory and post-inflammatory cytokines. Furthermore, un-denatured whey protein provides proline, which is important factor that assists in collagen production [44, 45] and aids in the normal development of collagen fibrils in a wounded region. It is accepted that un-denatured whey proteins can accelerate wound healing in animals with experimentally induced diabetes.

In addition to decreasing lipid peroxidation and increasing lymphocyte proliferation and Ig production, glutathione—which is abundant in un-denatured whey protein—also increases the numbers of mast cells and fibroblasts, which take part in remodeling.

Whey protein for medical use can be obtained from different animals. Camel milk in particular has been studied for this purpose. Camel-milk-extracted whey protein enhances normal inflammatory responses during cutaneous wound healing in experimentally induced diabetic rats, thus confirming the essential role of inflammation in the healing process [46]. After oral supplementation with whey protein, diabetic mice and rats have been tested for B- and T-lymphocyte functions [47]. Supplemented animals display enhanced cell proliferation and chemotactic capacity; these functions are associated with high levels of CCL21, CXCL12, MIP1a, MIP2, KC, CXCL3 and TGF-ß in the whey extracts [47, 48]. Providing a whey protein-enriched diet to diabetic animals also favors the rescue from apoptosis of long-lived macrophages present in wounds and an acceleration of the healing and closure of diabetic wounds [49]. Furthermore, a whey protein-enriched diet decreases the levels of ß-defensins 2 and 3 associated with increased free radicals and diminished glutathione caused by experimental diabetes [50].

In summary, diabetes-induced ulcers, at least in experimental models, display impair profiles of pro-inflammatory/anti-inflammatory factors. This phenomenon is associated with a delay in the resolution phase of the healing process because aberrant messages are sent to T- and B-lymphocytes and macrophages, thereby impairing re-epithelialization and remodeling (which is normally carried out by platelets, macrophages, epitheliocytes and fibroblasts and represents the final phase of healing-associated physiological inflammation). Oral supplementation with appropriate amino acids that have a strong tropism for immune cells involved in inflammation and healing (such as lymphocytes and macrophages) has been shown to reverse the delay in closing experimentally inflicted wounds mimicking diabetic ulcers. Whey proteins, especially those from camel milk, have interesting potential in treating patients suffering diabetic ulcers. Whey proteins are available or can be made available in a native form in a number of countries and can be synthesized, hopefully, at a reasonable price. This may be an interesting avenue to complement the local treatment of wounds.

### Topical (honey) bee venom and propolis constituents to accelerate wound healing in diabetes

#### Bee propolis and wound healing properties

Many facets of wound healing under redox control require a delicate balance between oxidative stress and antioxidants. Whereas normal physiological wound healing depends on low levels of ROS and oxidative stress, overexposure to oxidative stress leads to impaired wound healing. Antioxidants have been postulated to help control wound oxidative stress and thereby accelerate wound healing. Many antioxidants are available over the counter or by prescription, but only one, Medihoney™, is FDA-approved specifically to treat wound healing [51, 52]; this product is made from honey, as its name implies. However, there is a lack of understanding regarding the molecular mechanisms of action for such honey-based, anti-oxidative topical gels.

There is an increasing interest in the use of natural products in modern medicine as part of disease and patient management. Natural products have been widely used to dissect the basic mechanisms underlying fundamental questions in the life sciences and also as clinical therapeutics. Bee products are natural and have diverse applications in medical fields for the treatment of various diseases. The identification of bee products that may enhance skin repair can contribute to a better understanding of the wound healing process and generate a new strategy to combat chronic wounds. Bee venom and propolis, which possess considerable antioxidant capacities, are important components of bee-based products. Propolis has been reported to have a broad spectrum of biological and pharmacological properties, including antimicrobial, antioxidant, anti-inflammatory, immunomodulatory [53, 54], antiulcer, hepatoprotective, cardioprotective, and neuroprotective actions [55, 56]. It is important to note that propolis has been found to exert profound anti-infective actions and has been used in topical gels and in dentistry [57]. However, the chemical composition and beneficial properties of propolis vary greatly depending on phytogeographical area, seasonal collection time, and botanical sources. Depending on its exact composition (and origin), propolis may also exhibit powerful local antibiotic and antifungal properties [58–61].

Propolis possesses remarkable anti-cancer properties [62–64]. Experimental studies in cancer have revealed specific modes of actions of propolis. Propolis induces cell cycle arrest and apoptosis and decreases the expression of growth and transcription factors (including NF-κB) in pre-clinical models of human breast cancer. Notably, caffeic acid phenethyl ester down-regulates the *mdr-1* gene, which is considered responsible for the resistance of cancer cells to certain chemotherapeutic agents.

In total, more than 300 constituents have been identified in propolis samples, whereas the biological activity of propolis has been mainly attributed to a handful of substances, including flavonoids, terpenes, caffeic acid, ferulic acid, cumaric acid and esters. Propolis is characterized by many diverse activities, but only some of these activities have been substantiated by clinical and experimental evidence [65].

Propolis is believed to decrease tissue degeneration and has been proven to be effective in treating skin burns [66, 67].

With specific reference to wound healing, propolis has recently been demonstrated to accelerate wound healing in different animal models that mimic diabetic wounds [68, 69]. We (and others) have sought to explore the mechanisms by which propolis can ameliorate the diabetic condition. Propolis constituents and active metabolites had been well characterized in terms of chemistry [70]. The oral supplementation of diabetic mice with propolis has been found to restore the proliferation and chemotactic capacity of B- and T-lymphocytes toward chemokines by interfering with the lipid inflammatory pathway [71]. The topical application of propolis has further been shown to enhance the cutaneous wound healing of diabetic ulcers experimentally induced in mice by promoting TGF-ß/SMAD-mediated collagen production [72]. Therefore, propolis may have two potential properties in diabetes: *per os* to modify lipid dysregulation and locally to accelerate tissue remodeling. This possibility remains to be further explored through studies including randomized controlled trials.

#### Bee venom and wound healing

In addition to the honey derivative propolis, there has traditionally been interest in another honeybee product: venom. Bee venom therapy is one of the most traditional complementary and alternative therapies and has long been believed to be effective in the treatment of many diseases, including rheumatic arthritis, bursitis, tendinitis, shingles (herpes zoster), multiple sclerosis and other autoimmune diseases, wounds, gout, burns, infections, and even cancer. This approach is primarily expected to relieve pain and inflammation and restore normal functions [73–75]. Although bee venom therapy may be a promising alternative for the treatment of chronic pain, in contrast to honey propolis, it has not been approved as being effective and safe by the worldwide food and drug authorities.

Honeybee venom is composed of melittin, phospholipase A2, apamin, mast cell degranulating peptide and several bioactive amines, such as histamine and epinephrine, among other components. Melittin and phospholipase A2 are the two major components of bee venom (40–60% and 15–20%, respectively [76]). These components are generally thought to play an important role in the induction of the irritation and allergic reactions associated with bee stings. The injection of bee venom has been reported to evoke hyperalgesia and a sharp pricking sensation followed by tonic pain lasting for a few minutes up to 1–2 h.

However, there is conflicting evidence in the literature to suggest that bee venom may also exert anti-inflammatory and anti-nociceptive effects [77, 78]. One group claiming that melittin has certain anti-inflammatory effects has provided interesting evidence of an influx of cytokines and nitric oxide caused by bee venom, which have been postulated to play important roles in mediating the cell recruitment and activation necessary to balance inflammation/anti-inflammation and repair tissue damage [79]. Recently, it has further been demonstrated that bee venom can experimentally accelerate wound healing in diabetic mice by suppressing activating transcription factor-3 (ATF-3) and inducible nitric oxide synthase (iNOS)-mediated oxidative stress. Evidence has also been obtained that bee venom can help recruit bone marrow-derived endothelial cells, thus accelerating re-epithelialization and tissue remodeling [80].

## Conclusions

There are distinct avenues to pursue when considering certain ‘gifts’ of ‘nature’ as potential universal drugs. One must decipher mode(s) of action at the cellular and molecular levels (most natural products exert antioxidant activities that have strong inflammatory/anti-inflammatory potential). One must also understand how to use these ‘gifts’ to provide care and cure and not cause harm. In other words, it must be determined that the benefit/risk is highly favorable. Finally, one must test the products in registered trials and evaluate them in terms of their benefits compared to commonly accepted treatments. The use of well-characterized ‘natural’ antioxidants has interesting potential in countries with developing economies because these products can be made available at a low cost if the natural resource is abundant. Products of natural origin must thus be taken into consideration along with current conventional therapies in regenerative medicine [81] as well as novel cell therapies, such as promising mesenchymal cell therapy [82, 83].
